# First Report of *Anaplasma phagocytophilum* in Galapagos: High Prevalence in Dogs and Circumstantial Evidence for the Role of *Rhipicephalus linnaei* as Vector

**DOI:** 10.1155/tbed/5542334

**Published:** 2025-07-03

**Authors:** Carla Andreea Culda, Luciana Cătălina Panait, Cristina Daniela Cazan, Hein Sprong, Rommel Lenin Vinueza, Diego Páez-Rosas, Renato Leon, Andrei Daniel Mihalca

**Affiliations:** ^1^Department of Parasitology and Parasitic Diseases, University of Agricultural Sciences and Veterinary Medicine of Cluj-Napoca, Cluj-Napoca, Romania; ^2^Department of Public Health and Food Hygiene, University of Agricultural Sciences and Veterinary Medicine of Cluj-Napoca, Cluj-Napoca, Romania; ^3^Centre for Infectious Disease Control (CIb), National Institute for Public Health and the Environment (RIVM), Bilthoven, Netherlands; ^4^School of Veterinary Medicine, Cumbayá, San Francisco University of Quito USFQ, Quito, Ecuador; ^5^Laboratory of Medical Entomology and Tropical Medicine LEMMT, San Francisco University of Quito USFQ, Quito, Ecuador; ^6^College of Biological and Environmental Sciences COCIBA, San Francisco University of Quito USFQ, Quito, Ecuador; ^7^Galapagos Science Center GSC, USFQ and UNC-Chapel Hill, San Cristóbal Island, Galápagos, Ecuador; ^8^Galápagos Conservancy Foundation, Galapagos Conservancy, Santa Cruz Island, Galápagos, Ecuador; ^9^Galápagos National Park Directorate, San Cristóbal Technical Operational Unit, San Cristóbal Island, Galapagos, Ecuador

**Keywords:** *Anaplasma platys*, *Anaplasma phagocytophilum*, canine anaplasmosis, *Rhipicephalus linnaei*, tick-borne fever

## Abstract

The current study investigates the presence and prevalence of *Anaplasma* species in dogs from the Galapagos Islands, focusing on the potential vectorial role of *Rhipicephalus linnaei* in the transmission of these pathogens. Blood samples were collected from 1221 dogs across four islands, with tick collections for morphological and genetic identification. The results revealed a significant molecular prevalence of *Anaplasma phagocytophilum* (20.3%), predominantly in Santa Cruz (35.16%) and Isabela (18.9%), while *A. platys* was identified in 2.9% of samples. Genetic analysis identified the presence of *A. phagocytophilum* ecotype I, aligning more closely with European strains. Furthermore, *R. linnaei* was confirmed as the only tick species associated with dogs, suggesting its role as a vector for both *A. phagocytophilum* and *A. platys*. This study marks the first molecular confirmation of these pathogens in the Galapagos, contributing with important insights into the epidemiology of tick-borne diseases in this ecosystem. The findings highlight the need for improved surveillance and control to reduce the risk and further spread of these tick-borne diseases.

## 1. Introduction

Intracellular gram-negative bacteria of the genus *Anaplasma* are the agents of tick-borne diseases known generally as anaplasmoses, which can affect both human and animal health [1–3]. *Anaplasma phagocytophilum* is one of the most common zoonotic pathogens that is widespread worldwide [4–8]. Therefore, the zoonotic potential of this pathogen could be associated with its ability to cross species [3, 8, 9]. Another species, *Anaplasma platys*, is also known to infect humans, dogs, and other wild and domestic carnivores [7, 10–12] causing canine cyclic thrombocytopenia.

The clinical outcome of the *Anaplasma* infections depends on the bacterial species and genotype, as well as on the host species and its comorbidities, presenting a complex diagnostic challenge [13, 14]. *Anaplasma phagocytophilum* infects especially neutrophils, leading to a decrease in the number of neutrophils and leukocytes, resulting in suppression of the host body's immune system [15]. This disease exhibits acute and nonspecific symptoms such as fever, anorexia, lethargy, lameness, polyarthritis, and cytoplasmic inclusions in neutrophils [3, 7, 16, 17]. Clinical manifestations caused by *A. platys* in dogs include temperature fluctuations, changes in pulse rate, breathing difficulties, loss of appetite, weight loss, nasal discharge, and vomiting, all of which are associated with damage to the immune system [7, 15, 16, 18].

All *Anaplasma* species are transmitted by ticks [8, 19]. However, despite their almost worldwide distribution, the vectorial role of certain tick species is poorly understood in many parts of the world. *Anaplasma phagocytophilum* was found in various tick species, but only a few tick species have been confirmed as competent vectors, such as *Ixodes ricinus*, *I. persulcatus*, *I. scapularis*, *Haemaphysalis longicornis*, and *H. concinna* [20]. However, many reports show the presence of *A. phagocytophilum* in areas were these generally recognized vectors are absent, including several tropical and subtropical areas such as Madeira Island [21], Sardinia [22], Vancouver Island [23], Japan [24], China [25], Caribbean Islands [26], Algeria [27], Costa Rica [28], and Ethiopia [29]. In the case of *A. platys*, the main incriminated vectors were traditionally referred to as *R. sanguineus* s.l.; however, since the recent delimitation of species within this group [30–33], and the possibly different vectorial role [17, 34–36], new data are essential to understand disease transmission risk by each of these species.

In this new context, the aim of the current study was to verify the presence of *Anaplasma* species in the blood of dogs from the Galapagos and provide an epidemiological context to indicate the potential role of *Rhipicephalus linnaei* in the transmission cycle of these pathogens.

## 2. Materials and Methods

### 2.1. Sampling

**Figure 1:**
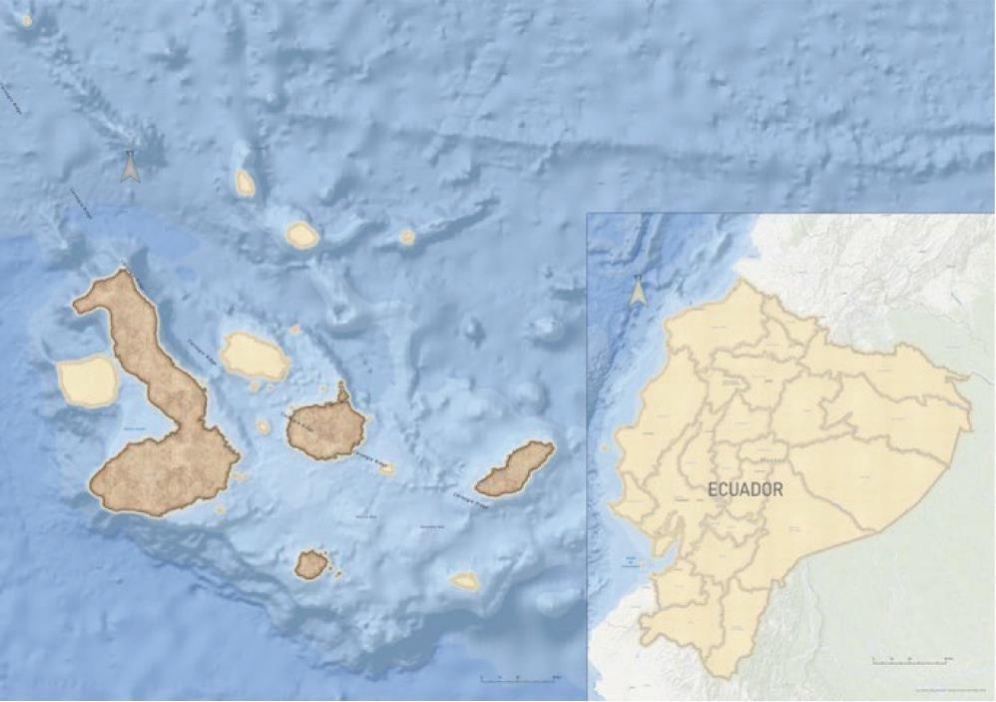
Sampling collection area (dark brown: Islands).

The Galapagos Islands, a group of young volcanic islands situated around the equator about 900 km west of South America (Figure 1), present different types of habitats on each island [37]. Almost all the islands, especially the inhabited ones (San Cristóbal, Isabela, Santa Cruz, and Floreana), feature a range of habitat types at different altitudes, including jungles, dry woodlands, and swampy moorland [37, 38]. The annual temperature ranges from 31 to 26°C, creating a tropical climate that is favorable for ticks year-round [39].

**Figure 2:**
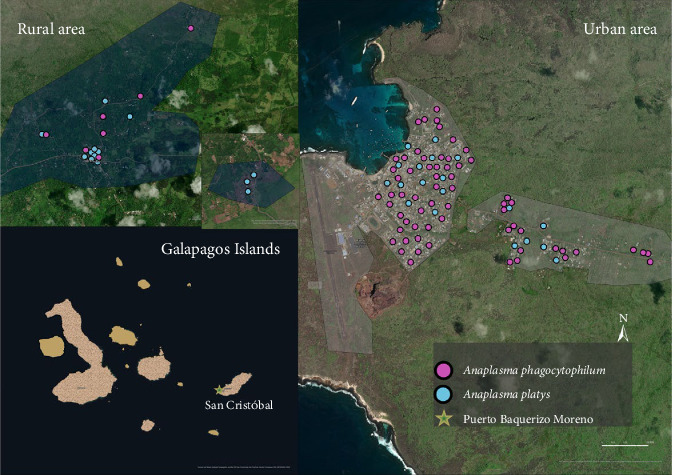
The spatial distribution of positive dogs on San Cristóbal Island.

**Figure 3:**
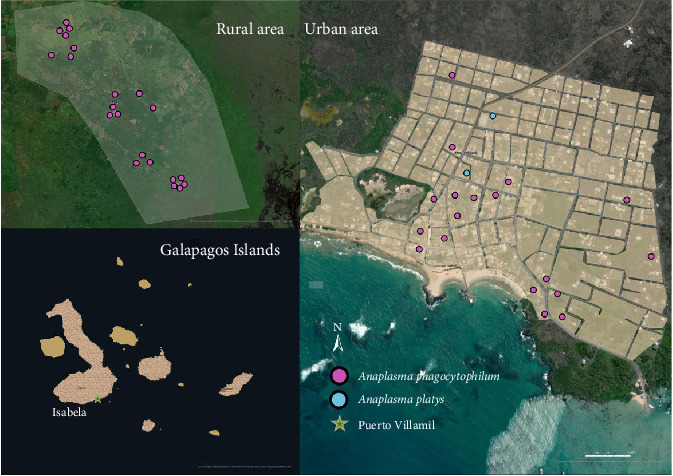
The spatial distribution of positive dogs on Isabela Island.

**Figure 4:**
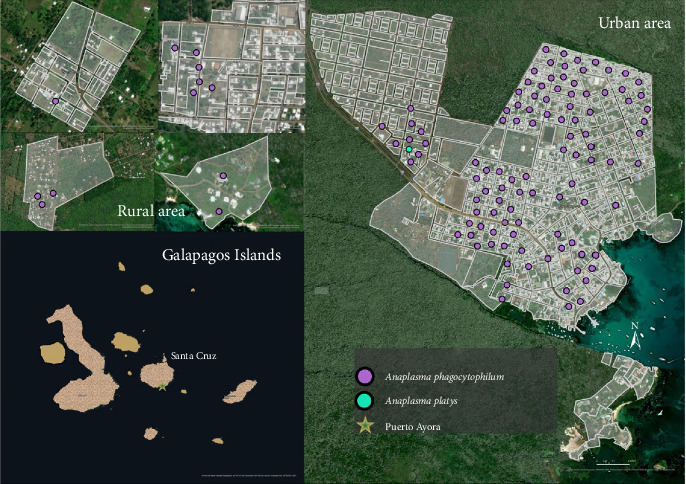
The spatial distribution of positive dogs on Santa Cruz Island.

**Figure 5:**
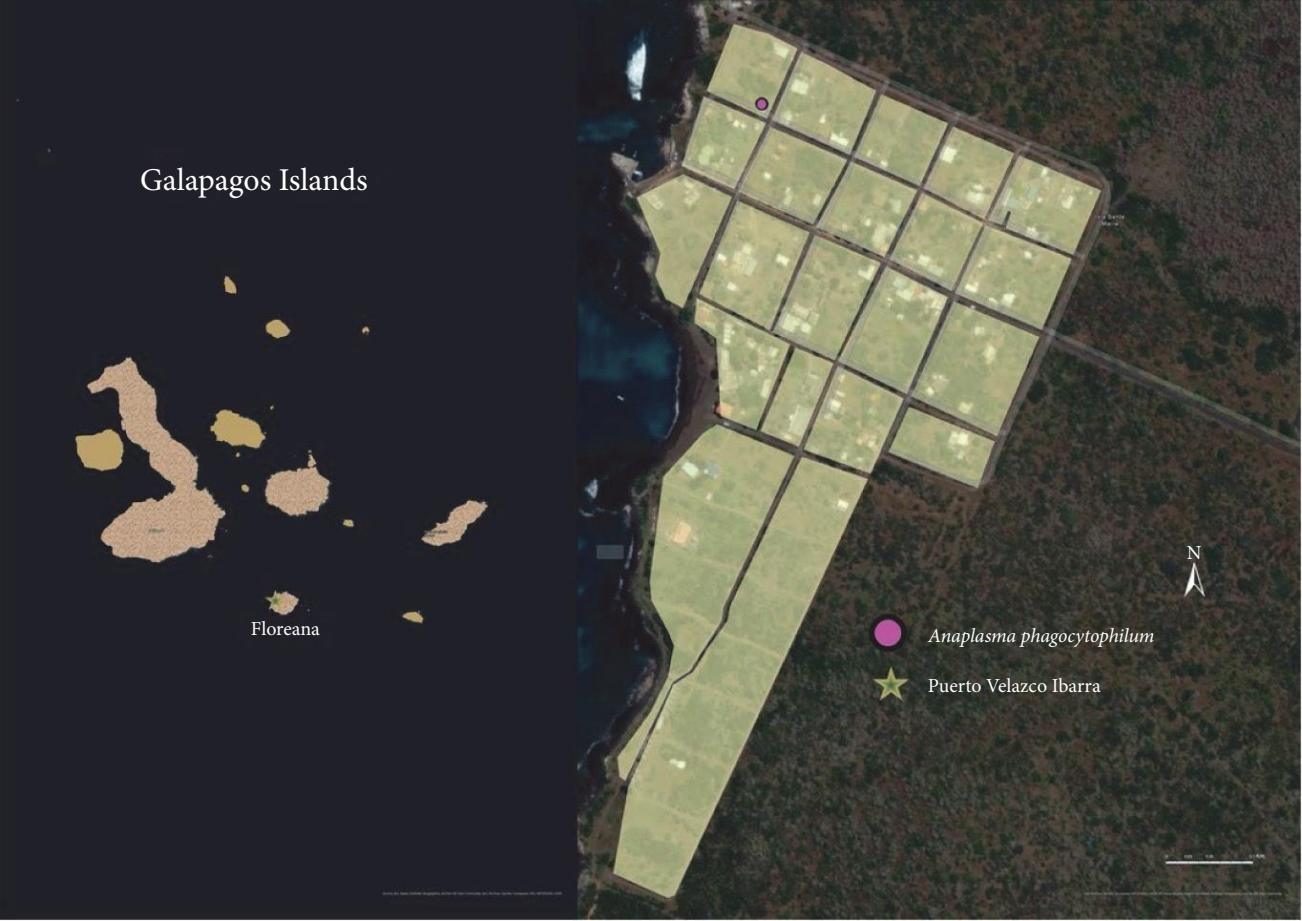
The spatial distribution of positive dogs on Floreana Island.

Blood samples and ticks from dogs were collected on four islands (San Cristóbal, Isabela, Santa Cruz, and Floreana; Figure 1). A total of 1221 owned dogs were sampled during 2021 and 2022 [40, 41]. The sampling was conducted in urban areas defined by different numbers of neighborhoods within the ports of each inhabited island. These areas include Puerto Baquerizo Moreno in San Cristobal, Puerto Ayora in Santa Cruz, Puerto Villamil in Isabela, and Puerto Velazco Ibarra in Floreana. In contrast, rural areas are defined as any other localities not fitting into urban (Figures 2–5).

In addition, for the purpose of this study, ticks were randomly collected from 90 dogs (52 males and 38 females) on the same islands. Ticks were stored in 70% ethanol at ambient temperature until further analysis.

### 2.2. DNA Extraction From Blood and PCR Amplification

Genomic DNA (gDNA) was extracted as mentioned in Culda et al. [41] using a specific kit, DNeasy Blood, and Tissue Kit (Qiagen, Germany), according to the instructions. The presence of *Anaplasma* spp. was evaluated by conventional and nested PCR amplifying fragments of the *groEl* gene of *A. phagocytophilum*, and for *A. platys* used two different PCR protocols with amplifying fragments of *rrs* gene and *gltA* gene (Table 1).

The amplification profile for *A. phagocytophilum* involved two rounds of amplification, as outlined by Alberti et al. [22]. Meanwhile, amplification of specific DNA for *A. platys* bacteria was conducted using the protocol and thermal amplification profile outlined by Mathew et al. [42]. All positive samples obtained for the*16S rRNA* gene of *A. platys* were retested for the *gltA* gene. Each amplified reaction included both a negative and a positive control to assess the reaction's quality and the possibility of cross contamination. The positive control consisted of DNA confirmed with the targeted species, and the negative control consisted of the reaction mixture with ultrapure water instead of DNA.

The amplicon obtained from the positive samples was purified using a DNA Fragments Extraction Kit (Geneaid Biotech Ltd., New Taipei City, Taiwan), followed by sequencing (Macrogen Europe, Amsterdam, and the Netherlands). The chromatographs of the sequences were assembled, analyzed, and the primer sites were trimmed in Bionumerics v.7.6 (Applied Maths, Sint-Martens-Latem, and Belgium). The DNA sequences were subjected to used BLAST analysis to identify the species and compare them to the relevant stored sequences in GenBank (Supporting Information 1).

### 2.3. Morphological Identification and DNA Extraction From Ticks

**Figure 6:**
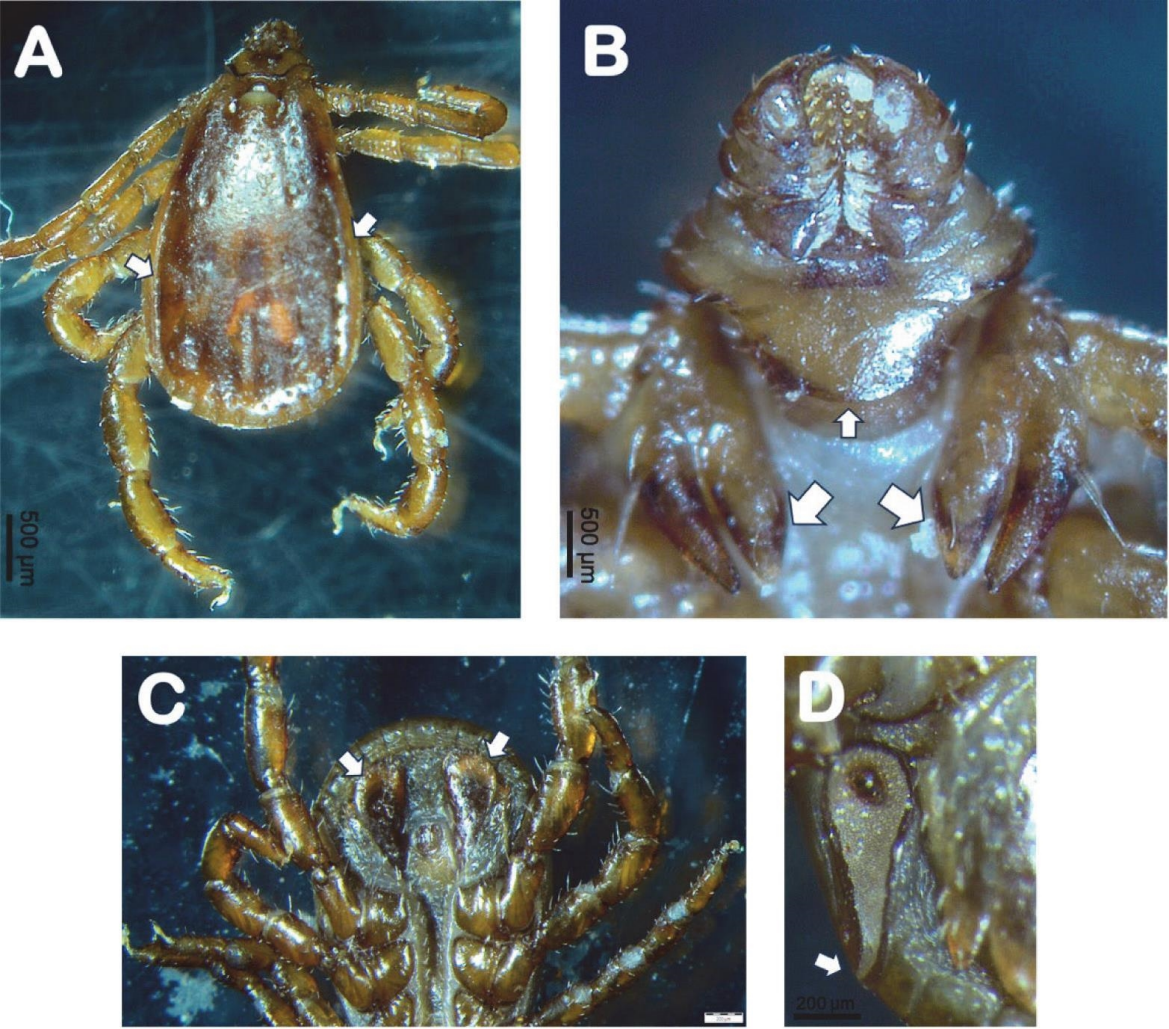
*Rhipicephalus linnaei* adult male collected from a dog in Galapagos islands. (A) The dorsal view shows a pear-shaped form characterized by a scutum featuring pronounced lateral grooves (arrows); (B) ventral view of the anterior part of the body showing coxa I with two long triangular spurs (large arrows) and the smooth basis capitulum with a mildly convex posterior margin (small arrow), and characteristic palps and hypostome [32]; (C) adanal shields have subtriangular shape (arrows); (D) spiracular plates showing characteristic shape, with the internal margin slightly concave in its anterior part, while the posterior part is rounded and without a spur (arrow) [32].

**Figure 7:**
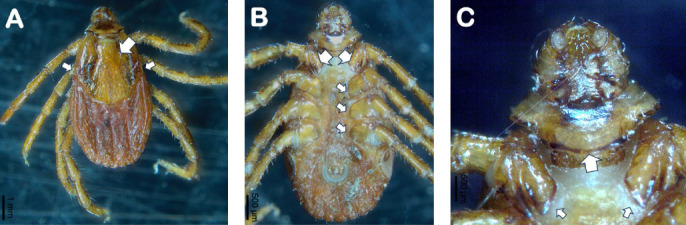
*Rhipicephalus linnaei* adult female collected from a dog in Galapagos Islands. (A) Dorsal view showing an inornate scutum, widest at the level of eyes (arrows), and overall brown coloration of the body. Cervical grooves form a bow-shaped depression together with the lateral grooves (large arrow); (B) ventral view with showing the typical morphology of coxae: coxa I has two long triangular spurs (large arrows; details also in C) and coxae II–IV (arrows) each with a short triangular external spur; (C) ventral view showing details of coxa 1 (arrows) and basis capituli (large arrow) with a blunt and short hypostome, with 3/3 dental formula.

All ticks were morphologically identified to the genus or species level using a stereo microscope (OLYMPUS SC180) and various taxonomic keys [32, 44–46]. Morphologically important features were photographed (Figures 6 and 7).

DNA extraction was conducted to support the morphological identification of collected ticks. Eighteen individual ticks were randomly selected in different phases of development (nymphs, males, and females) from each island (Table 2). All ticks were allowed to dry in a sterile 1.5 mL Eppendorf tube on a heat block. Dried ticks were used for the isolation of gDNA by the conventional DNeasy Blood and Tissue Kit (Qiagen, Germany). After incubating the protein lysis with proteinase K overnight, the liquid phase was used to isolate gDNA following the manufacturer's instructions, while the exoskeleton was preserved in 70% ethanol.

### 2.4. Ecotype

A selection of *groEL* sequences from a previously compiled database were compared with the sequences from this study [47]. A search for more recent *A. phagocytophilum* isolates, which had a high similarity with the sequences from this study were added. A subset of isolates with *groEL* sequences that spanned a longer fragment with a higher genetic resolution (LF), from 589 to 1118 (530) bp, was extracted for further analyses. After this selection, the dataset for ecotyping consisted of approximately 1640 field isolates. For this, a multiple alignment based UPGMA tree was generated in BioNumerics v.7.6 [48].

### 2.5. PCR Amplification for Tick Identification

Genetic analysis was conducted by amplifying the mitochondrial genes *cox1*, *16S rRNA*, and *12S rRNA* to clarify species status, *R. linnaei* (Table 3) [32].

### 2.6. PCR Amplification for Testing the Presence of Anaplasmosis in Ticks

All ticks, including larvae, nymphs, males, and females, were individually tested for the presence of bacterial species belonging to the family Anaplasmataceae using primers targeting the *groEL* and *gltA* genes [22, 43].

## 3. Statistical Analysis

Statistical analysis was performed using the EpiTools software. Odds ratios (ORs), 95% confidence intervals (CIs), and *p*-values were calculated for epidemiological statistical analysis.

## 4. Results

**Figure 8:**
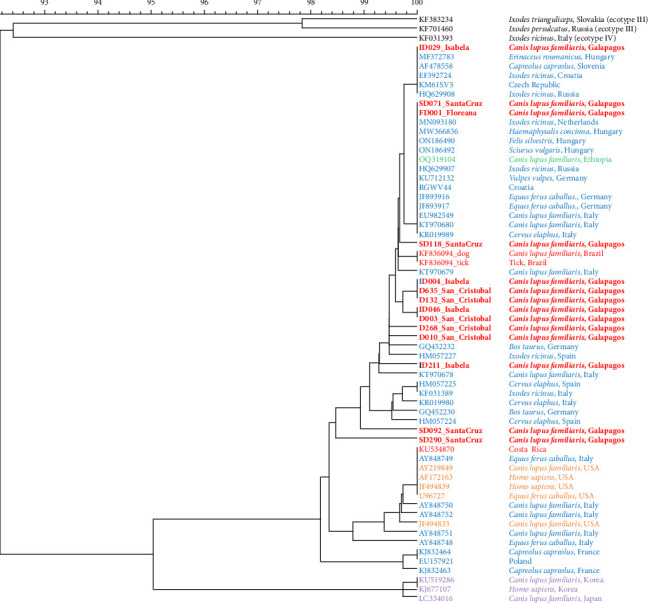
Phylogenetic trees for the *groEL* gene. The tree shows the phylogenetic relationships among the *groEL* gene sequences obtained from the studies. Related sequences are clustered together, as indicated next to the branches (red: south and central America; blue: Europe; green: Ethiopia; orange: North America; purple: Asia; sequences obtained in this study are in bold).

Overall, 247 out of 1221 blood samples (20.3%) were positive for *A. phagocytophilum* (Table 4). The DNA sequences revealed similarity (98.16%–100%) with the available DNA sequences of *A. phagocytophilum* in NCBI GenBank (Supporting Information 1). The obtained sequences can be found in Supporting Information 2. The prevalence of *A. phagocytophilum* in domestic dogs by island was: San Cristóbal (11.8%; 71/603; 95% CI: 9.2–14.4), Isabela (18.9%; 40/212; 95% CI: 13.6–24.1), Santa Cruz (35.16%; 135/384; 95% CI: 30.4–40), and Floreana (4.5%; 1/22; 95% CI: 0–13.3; Figures 2–5). All 14 *groEL* sequences from the islands clustered with *A. phagocytophilum* ecotype I originating from Europe, but not with *A. phagocytophilum* from North America or Asia (Figure 8).

The *16S rRNA* sequence of *A. platys* DNA was identified in 35 (2.9%) blood samples (Table 5). The prevalence of *A. platys* identified on the islands was as follows: San Cristóbal (5.4%; 32/603; 95% CI: 3.5–7.1), Isabela (1%; 2/212; 95% CI: 0–2.2), Santa Cruz (0.3%; 1/384; 95% CI: 0–0.7), and Floreana 0%;Figures 2–5. Additionally, the sequence analysis revealed high similarity (99.72%–100%) with various other sequences stored in GenBank (Supporting Information 3). The positive samples were retested for the *gltA* gene, and the sequences showed 99.73%–100% matching with sequences from GenBank (Supporting Information 3). The sequences obtained from the positive samples were deposited in the GenBank international database under the Accession No. PV539461–PV539462 and can also be found in Supporting Information 4.

A total of 463 ticks were collected from 90 dogs (Table 6). Out of these, 250 were identified to species level, while 213 were identified only to genus level due to extreme engorgement. All ticks identified to species level were morphologically consistent with *Rhipicephalus linnaei*. The specimens identified to genus level were all assigned to genus *Rhipicephalus*. For the 18 specimens randomly selected for molecular confirmation, the BLAST analysis revealed a 99%–100% similarity to *R. linnaei* sequences in GenBank (Supporting Information 5). The sequences obtained were deposited in the GenBank under the Accession No. (PV533807–PV533810; PV533889–PV533891; PV536164–PV536167).

## 5. Discussion

To our knowledge, this is the first molecular confirmation of *A. phagocytophilum* in Galapagos Archipelago, and the first report of *A. platys* in dogs from Santa Cruz, San Cristóbal, and Floreana Islands (Table 7). Dogs over 10 years old appeared to have higher infection rates of *A. phagocytophilum*, but this was not the case for *A. platys*. This could provide new information about the risk factors for *A. phagocytophilum*.

Our results show an unexpectedly high prevalence of *A. phagocytophilum* (20.3%) in dogs inhabiting the Galapagos Islands. This *Anaplasma* species has been reported from other tropical and subtropical regions with variable prevalence in dogs: 2.0%, Iran [58]; 27%, Mexico [59]; 7.6%, Northern California, USA [60]; 7.1%, Brazil [61]; 1.1%, Colombia [62]; 0.3%, Costa Rica [63]; and 0.4% - India [64], but also in other vertebrate hosts such as horses, domestic cats, wild carnivores, rabbits, rodents, vervet monkeys, baboons, and humans [65, 66] and in engorged ticks such as: *Rhipicephalus sanguineus* s.l. (most probably *R. linnaei*), in Egypt [67] and India [64]; or *Amblyomma cohaerens* in Ethiopia, [68] and unengorged *R. pulchellus* in Ethiopia [69], and *R. maculatus* in Kenya [70].

The presence of *A. platys*, although with lower prevalence, was not surprising, since it has also been reported in various tropical and subtropical regions, including oceanic islands [34, 71–77]. In Europe, the main reservoirs for *A. phagocytophilum* are wild and domestic ruminants [8], while the most important vectors are ticks of the *Ixodes* genus. The only ticks identified so far on dogs in Galapagos are the “brown dog ticks” or *R. sanguineus* s.l. [53, 78, 79]. However, until our study, no molecular or phenotypic evidence was available for the specific identity of this tick species. The recent work of Šlapeta et al. [32] has demonstrated that the *R. sanguineus* s.l. “tropical lineage” belongs to *R. linnaei*, followed by the designation of a neotype. In line with these current concepts, we were able to confirm the identity of the brown dog ticks from Galapagos as *R. linnaei*. Similarly, *R. linnaei* was also recently confirmed in Colombia and Mexico [36].

The specific vectorial role of *R. linnaei* has not been thoroughly investigated since the clarification of the identity of the so-called tropical linage. Some authors have suggested its potential role as a vector for *Rickettsia massiliae*, *A. phagocytophilum*, *Francisella tularensis*, *Theileria equi*, and *Ehrlichia canis* [36, 80–83]. Our results strongly suggest that *R. linnaei* acts as a vector for *A. phagocytophilum* and *A. platys*. Furthermore, based on our previous findings, [41] *R. linnaei* may also serve as a vector for *Babesia vogeli* and *Hepatozoon canis*. Furthermore, it is essential to conduct controlled laboratory experiments to test the vectorial capacity of *R. linnaei* to unequivocally demonstrate its role as a vector for these pathogens. The transmission cycle of these pathogens on the Galapagos Islands requires further evaluation, as there was no prior molecular or phenotypic evidence indicating the presence of *R. linnaei* before this study. Additional research will be necessary to investigate this aspect.

We confirmed that *A. phagocytophilum* from the Galapagos belongs to ecotype I. The sequences obtained from these islands are more closely related to those found in Europe than to those in Asia and North America. However, not all positive samples could be attributed to an ecotype, as sequencing was performed only on a subset of positive samples. This is not unusual, as Santos et al. [84] revealed a 100% match between the gene sequences obtained from dogs and ticks in Brazil with the strain isolated from dogs in Germany. A study Ethiopia revealed similarity between the DNA sequences of *A. phagocytophilum* from dogs with sequences from Europe [29] and Alberti et al. [22] found that *A. phagocytophilum* isolated from Sardinia, Italy, was closely related phylogenetically to strains in the United States. This geographical pattern cannot, unfortunately, answer the question of how and when these strains were introduced in different parts of the world, nor their true origin. This is also evident from phylogenetic analysis (Figure 6).

The prevalence data from the present study may be linked to inadequate preventive measures against ectoparasites, which exposes most dogs to the significant risk of developing vector-borne diseases [53]. During sampling, 90% of participants reported that they never or very rarely administered any type of medication or veterinary product to their dogs (Culda, pers. obs.). Furthermore, the frequency of dogs that tested positive for *Anaplasma* spp. is higher in urban areas than in rural areas, as noted in previous studies [53, 54].

## 6. Conclusion

The present study indicates that *R. linnaei* is present in the Galapagos Islands, and may act as a vector for *A. phagocytophilum* (ecotype 1) and *A. platys*. These results broaden the knowledge of the vectorial role of the species in the *R. sanguineus* s.l. group, as well as our data on the distribution of tick-borne pathogens in the Galapagos Archipelago. The global spread of *Anaplasma* spp. poses significant public health concerns.

## Figures and Tables

**Table 1:** Primers sequences used for each pathogen.

Pathogen (bp)	Primer name and sequence	Gene	Reference
*A. phagocytophilum*, 530	groEL1: ATGGTATGCAGTTTGATCGC	*groEl*	[22]
	groEL2: TTGAGTACAGCAACACCACCGGAA		
	groEL1n: GTCGAATTTGAAAATCCATAC		
	groEL2n: GTCCTGCTAGCTATGCTTTC		

*A. platys*, 349	EPLAT5: TTTGTCGTAGCTTGCTATGAT	*16S rRNA*	[42]
	EPLAT3: CTTCTGTGGGTTACCGTC		

*A. platys*	PlgltA1f: TTGGATATTGGGTAACGCTG	*gltA*	[43]
	PlgltA1r: CACTTCCTTCCGGGGTATACCAC		

**Table 2:** Number of ticks by stage and location which were genetically analyzed.

Islands	Nymphs	Males	Females
San Cristóbal	0	5	4
Isabela	1	2	1
Santa Cruz	0	2	1
Floreana	0	1	1

Total	1	10	7

**Table 3:** Primers sequences used for molecular characterization.

Primer name and sequence (5′–3′)	Gene	Reference
LCO1490: GGTCAACAAATCATAAAGATATTGG	*cox1*	[49, 50]
HCO2198: TAAACTTCAGGGTGACCAAAAAATCA		
S0725: TACTCTACTAATCATAAAGACATTGG		
S0726: CCTCCTCCTGAAGGGTCAAAAAATGA		

S0749: CTGCTCAATGATTTTTTAAATTGCTGTGG	*16S rRNA*	[49, 51]
S0750: TTACGCTGTTATCCCTAGAG		

S0738_T1B: AAACTAGGATTAGATACCCT	*12S rRNA*	[49, 52]
S0739_T2A: AATGAGAGCGACGGGCGATGT		

**Table 4:** The prevalence of *Anaplasma phagocytophilum* in dogs.

Variable	Category	No. of samples	Positive (%)	*p*-Value	OR	95% CI
Total	—	1221	247 (20.3)	—	—	18–22.5

Sex	Males	652	132 (20.3)	1	1.0021	17.2–23.3
	Females	569	115 (20.3)			17–23.6

Age	<1 year	149	8 (5.4)	0.0001*⁣*^*∗*^	NA	1.8–9
	1–4 years	648	120 (18.6)			15.6–21.5
	4–10 years	345	77 (22.4)			17.9–26.7
	>10 years	50	16 (32.0)			19–44.9
	Unknown age	29	0 (0)			0

Breed	Pure breed	385	79 (20.6)	0.9246	1.0265	16.5–24.6
	Mixed breed	836	168 (20.1)			17.4–22.8

Environment	Urban	1013	209 (20.7)	0.4979	1.1629	18.2–23.1
	Rural	208	38 (18.3)			13–23.5

Housing^a^	Outdoor	560	89 (15.9)	0.0005*⁣*^*∗*^	NA	12.9–18.9
	Indoor	231	64 (27.8)			21.9–33.5
	Outdoor and indoor	430	94 (21.9)			18–25.8

Free roaming	Yes	308	64 (20.8)	0.8448	1.0463	16.3–25.3
	No	913	183 (20.1)			17.5–22.6

Abbreviations: 95% CI, 95% confidence interval; NA, not applicable; OR, odds ratio.

^a^The lifestyle of the dogs.

*⁣* ^*∗*^Statistically significant.

**Table 5:** The prevalence of *Anaplasma platys* in dogs.

Variable	Category	No. of samples	Positive (%)	*p*-Value	OR	95% CI
Total	—	1221	35 (2.9)	—	—	1.9–3.8

Sex	Males	652	18 (2.8)	0.948	0.9219	1.5–4.3
	Females	569	17 (3)			1.6–4.4

Age	<1 year	149	8 (5.4)	0.001*⁣*^*∗*^	NA	1.8–9
	1–4 years	648	12 (1.9)			0.8–3.2
	4–10 years	345	9 (1.4)			0.9–4.3
	>10 years	50	2 (4.0)			0–9.4
	Unknown age	29	4 (13.8)			1.3–26.4

Breed	Pure breed	385	8 (2.1)	0.3492	0.6358	1–3.5
	Mixed breed	836	27 (3.2)			2.1–4.5

Environment	Urban	1013	31 (3.1)	0.5047	1.61	2–4.1
	Rural	208	4 (2)			0.6–3.8

Housing^a^	Outdoor	560	22 (7.2)	0.1188	NA	2.3–5.5
	Indoor	231	4 (1.8)			0.05–3.4
	Outdoor and indoor	430	9 (2.1)			1–3.5

Free roaming	Yes	308	10 (3.3)	0.791	1.1919	1.3–5.2
	No	913	25 (2.8)			1.7–3.8

Abbreviations: 95% CI, 95% confidence interval; NA, not applicable; OR, odds ratio.

^a^The lifestyle of the dogs.

*⁣* ^*∗*^Statistically significant.

**Table 6:** Developmental stages of ticks collected according to the location.

Islands	Larvae	Nymphs	Adults	No. of dogs
San Cristóbal	0	8	113	28
Isabela	18	12	268	53
Santa Cruz	0	0	19	7
Floreana	0	0	36	2

Total	18	20	436	90

**Table 7:** Prevalence of *Anaplasma* spp. in the Galapagos Islands reported in various studies.

Location	Prevalence (%)	Host	Method	Reference
Santa Cruz				
* Anaplasma* spp.	13.51	Dog	Antibody^a^	[53]
	24	Dog	Antibody^a^	[54]
	12.1	Dog	Antibody^a^	[55]
*A. platys*-like	46.7 (7/15)	Cattle	PCR^b^	[56]
*A. phagocytophilum*	0	Cattle	PCR	[56]
*A. phagocytophilum*	35.16	Dog	PCR	Present study
*A. platys*	0.3	Dog	PCR	Present study
Isabela				
*A. platys*-like	0	Cattle	PCR^b^	[56]
*A. platys*	1 (1 dog)	Dog	PCR	[57]
*Anaplasma* spp.	0	Cat	PCR	[57]
*A. phagocytophilum*	18.9	Dog	PCR	Present study
*A. platys*	1	Dog	PCR	Present study
San Cristóbal				
*A. platys*-like	44.4 (4/9)	Cattle	PCR^b^	[56]
*A. phagocytophilum*	11.8	Dog	PCR	Present study
*A. platys*	5.4	Dog	PCR	Present study
Floreana				
*A. phagocytophilum*	4.5	Dog	PCR	Present study
*A. platys*	0	Dog	PCR	Present study

^a^Commercial ELISA test (SNAP 4Dx Plus test).

^b^Genetic sequencing.

## Data Availability

All the data generated or analyzed during this study are included in this publication. The data that support the findings of this study are available in the supporting information of this article.

## Supporting Information

Supporting Information 1 BLASTn comparisons of the isolates from the current study with *A. phagocytophilum GroEL* gene sequences from GenBank (November, 2024)

Supporting Information 2 Sequences obtained for *A. phagocytophilum* GroEL gene

Supporting Information 3 BLASTn comparisons of the isolates from the current study with *A. platys* sequences from GenBank (June, 2024)

Supporting Information 4 Sequences obtained for *A. platys* 16S gene

Supporting Information 5 BLAST comparisons between the obtained sequences with *Rhipicephalus linnaei* from GenBank (August, 2024)
