# Supplementary material for: First Report of Anaplasma phagocytophilum in Galapagos: High Prevalence in Dogs and Circumstantial Evidence for the Role of Rhipicephalus linnaei as Vector
Source: Transbound Emerg Dis. 2025 Jul 3;2025:5542334. doi: 10.1155/tbed/5542334 (PMC12245514; doi:10.1155/tbed/5542334)
Supplement: Supporting Information 3 — BLASTn comparisons of the isolates from the current study with A. platys sequences from GenBank (June, 2024) [file 5542334.f3.docx]

BLAST comparisons between the obtained sequences with *Rhipicephalus linnaei* from GenBank (August/2024)

| **Isolate** | **Stage** | **Origin** | **Gene** | **Query cover** | **E-value** | **Percent identity^*^ (%)** | **Closest Match Accession Number** | **Host** | **Location** |
| --- | --- | --- | --- | --- | --- | --- | --- | --- | --- |
| D431 - B | F | San Cristóbal | 16S | 100 | 6E-122 | 99.20 | MF425981 | - | Angola |
| D431 - B | M | San Cristóbal | 16S | 100 | 1E-149 | 100 | MF425981 | - | Angola |
| D546 - B | F | San Cristóbal | 16S | 100 | 1E-149 | 99.66 | MF425981 | - | Angola |
| D546 - B | M | San Cristóbal | 16S | 100 | 8E-126 | 100 | MF425981 | - | Angola |
| ID048 | M | Isabela | 16S | 100 | 3E-150 | 100 | MF425981 | - | Angola |
| ID 140 | M rev | Isabela | 16S | 100 | 7E-85 | 100 | MF425981 | - | Angola |
| ID155 | N | Isabela | 16S | 94 | 4E-88 | 97.50 | PP919884 | *Canis lupus familiaris* | Mexico |
| SD073 | M | Santa Cruz | 16S | 100 | 3E-79 | 98.84 | MF425981 | - | Angola |
| SD114 | F | Santa Cruz | 16S | 94 | 5E-76 | 100 | OP352775 | *Canis lupus familiaris* | Croatia |
| SD114 | M forw | Santa Cruz | 16S | 93 | 7E-96 | 100 | OP352775 | *Canis lupus familiaris* | Croatia |
| SD114 | M rev | Santa Cruz | 16S | 100 | 1E-103 | 100 | MW429382 | *Canis lupus familiaris* | Fiji |
| FD009 | F | Floreana | 16S | 100 | 4E-93 | 98.99 | MF425981 | - | Angola |
| FD009 | F | Floreana | 16S | 100 | 2E-95 | 99.01 | MF425981 | - | Angola |
| FD009 | F | Floreana | 16S | 83 | 6E-31 | 84.87 | OP352775 | *Canis lupus familiaris* | Croatia |
| FD009 | M | Floreana | 16S | 99 | 4E-144 | 98.65 | MF425981 | - | Angola |
| D162 - A | M | San Cristóbal | 16S | 99 | 1E-88 | 98.45 | MF425981 | - | Angola |
| D162 - A | F | San Cristóbal | 16S | 100 | 3E-17 | 100 | OK353742 | *Capra hircus* | Cameroon |
| D234 - B | M | San Cristóbal | 16S | 100 | 6E-86 | 100 | MF425981 | - | Angola |
| D260 - C | F | San Cristóbal | 16S | 100 | 100 | 98.63 | MF425981 | - | Angola |
| D429 - B | M | San Cristóbal | 16S | 100 | 2E-28 | 93.68 | PP919885 | *Canis lupus familiaris* | Saudi Arabia |
| ID069 | F | Isabela | 16S | 100 | 2E-85 | 99.44 | ON510075 | *Canis lupus familiaris* | Nigeria |
| D431 - B | F forw | San Cristóbal | cox 1 | 100 | 0 | 99.64 | PP919883 | *Canis lupus familiaris* | Mexico |
| D431 - B | M | San Cristóbal | cox 1 | 100 | 0 | 99.52 | PP919884 | *Canis lupus familiaris* | Mexico |
| D546 - B | F | San Cristóbal | cox 1 | 100 | 0 | 99.52 | PP919884 | *Canis lupus familiaris* | Mexico |
| D546 - B | M | San Cristóbal | cox 1 | 100 | 0 | 99.36 | PP919884 | *Canis lupus familiaris* | Mexico |
| ID048 | M | Isabela | cox 1 | 100 | 0 | 99.53 | PP919884 | *Canis lupus familiaris* | Mexico |
| ID 140 | M | Isabela | cox 1 | 100 | 0 | 99.36 | PP919884 | *Canis lupus familiaris* | Mexico |
| ID155 | N | Isabela | cox 1 | 100 | 0 | 99.22 | PP919884 | *Canis lupus familiaris* | Mexico |
| SD073 | M | Santa Cruz | cox 1 | 100 | 0 | 99.52 | PP919884 | *Canis lupus familiaris* | Mexico |
| SD114 | F | Santa Cruz | cox 1 | 99 | 0 | 99.22 | PP919884 | *Canis lupus familiaris* | Mexico |
| SD114 | M | Santa Cruz | cox 1 | 100 | 0 | 99.07 | PP919884 | *Canis lupus familiaris* | Mexico |
| SD114 | M forw | Santa Cruz | cox 1 | 100 | 0 | 99.15 | PP919884 | *Canis lupus familiaris* | Mexico |
| SD114 | M rev | Santa Cruz | cox 1 | 100 | 0 | 99.65 | MW429383 | *Canis lupus familiaris* | Fiji |
| FD009 | F | Floreana | cox 1 | 100 | 0 | 99.52 | PP919884 | *Canis lupus familiaris* | Mexico |
| FD009 | F rev | Floreana | cox 1 | 98 | 0 | 99.66 | PP919884 | *Canis lupus familiaris* | Mexico |
| FD009 | M | Floreana | cox 1 | 100 | 0 | 99.52 | PP919884 | *Canis lupus familiaris* | Mexico |
| D162 - A | M | San Cristóbal | cox 1 | 100 | 0 | 99.52 | PP919884 | *Canis lupus familiaris* | Mexico |
| D234 - B | M | San Cristóbal | cox 1 | 100 | 0 | 99.20 | PP919884 | *Canis lupus familiaris* | Mexico |
| D260 - C | F | San Cristóbal | cox 1 | 100 | 0 | 99.36 | PP919884 | *Canis lupus familiaris* | Mexico |
| D429 - B | M | San Cristóbal | cox 1 | 100 | 0 | 99.36 | PP919884 | *Canis lupus familiaris* | Mexico |
| ID069 | F | Isabela | cox 1 | 100 | 0 | 99.36 | PP919883 | *Canis lupus familiaris* | Mexico |
| D431 - B | F | San Cristóbal | 12S | 99 | 1E-174 | 99.59 | PP919884 | *Canis lupus familiaris* | Mexico |
| D431 - B | F rev | San Cristóbal | 12S | 100 | 9E-131 | 99.89 | PP919884 | *Canis lupus familiaris* | Mexico |
| D431 - B | M | San Cristóbal | 12S | 100 | 2E-178 | 99.71 | PP919884 | *Canis lupus familiaris* | Mexico |
| D546 - B | F | San Cristóbal | 12S | 100 | 4E-175 | 99.14 | PP919884 | *Canis lupus familiaris* | Mexico |
| D546 - B | M | San Cristóbal | 12S | 100 | 1E-179 | 99.71 | PP919884 | *Canis lupus familiaris* | Mexico |
| ID048 | M | Isabela | 12S | 100 | 2E-178 | 99.71 | PP919884 | *Canis lupus familiaris* | Mexico |
| ID 140 | M | Isabela | 12S | 100 | 5E-174 | 98.86 | PP919884 | *Canis lupus familiaris* | Mexico |
| ID155 | N | Isabela | 12S | 100 | 2E-167 | 97.71 | MW429383 | *Canis lupus familiaris* | Fiji |
| ID155 | N forw | Isabela | 12S | 100 | 2E-137 | 99.64 | MW429383 | *Canis lupus familiaris* | Fiji |
| SD073 | M | Santa Cruz | 12S | 99 | 4E-180 | 99.44 | PP919884 | *Canis lupus familiaris* | Mexico |
| SD114 | F | Santa Cruz | 12S | 98 | 4E-180 | 99.72 | PP919884 | *Canis lupus familiaris* | Mexico |
| SD114 | M | Santa Cruz | 12S | 100 | 0 | 99.72 | PP919884 | *Canis lupus familiaris* | Mexico |
| SD114 | M rev | Santa Cruz | 12S | 100 | 4E-165 | 100 | PP919884 | *Canis lupus familiaris* | Mexico |
| FD009 | F | Floreana | 12S | 99 | 6E-178 | 100 | PP919884 | *Canis lupus familiaris* | Mexico |
| FD009 | M | Floreana | 12S | 100 | 4E-180 | 99.72 | PP919884 | *Canis lupus familiaris* | Mexico |
| FD009 | M rev | Floreana | 12S | 100 | 1E-154 | 100 | PP919884 | *Canis lupus familiaris* | Mexico |
| D162 - A | M | San Cristóbal | 12S | 100 | 5E-179 | 99.71 | PP919884 | *Canis lupus familiaris* | Mexico |
| D162 - A | M rev | San Cristóbal | 12S | 100 | 1E-154 | 100 | PP919884 | *Canis lupus familiaris* | Mexico |
| D162 - A | F | San Cristóbal | 12S | 100 | 2E-177 | 99.15 | PP919884 | *Canis lupus familiaris* | Mexico |
| D162 - A | F rev | San Cristóbal | 12S | 100 | 7E-152 | 100 | PP919884 | *Canis lupus familiaris* | Mexico |
| D234 - B | M | San Cristóbal | 12S | 100 | 2E-178 | 99.15 | PP919884 | *Canis lupus familiaris* | Mexico |
| D234 - B | M forw | San Cristóbal | 12S | 100 | 3E-151 | 99.67 | PP919884 | *Canis lupus familiaris* | Mexico |
| D234 - B | M rev | San Cristóbal | 12S | 100 | 9E-141 | 99.63 | PP919884 | *Canis lupus familiaris* | Mexico |
| D260 - C | F | San Cristóbal | 12S | 100 | 0 | 99.72 | PP919884 | *Canis lupus familiaris* | Mexico |
| D260 - C | F rev | San Cristóbal | 12S | 100 | 1E-144 | 100 | PP919884 | *Canis lupus familiaris* | Mexico |
| D429 - B | M rev | San Cristóbal | 12S | 100 | 4E-149 | 99.66 | PP919884 | *Canis lupus familiaris* | Mexico |
| ID069 | F | Isabela | 12S | 100 | 2E-178 | 99.71 | PP919884 | *Canis lupus familiaris* | Mexico |

^*^the percentage of the nucleotides that are the same between the two sequences (M – male; F – female; N – nymph)
