# Supplementary material for: First Report of Anaplasma phagocytophilum in Galapagos: High Prevalence in Dogs and Circumstantial Evidence for the Role of Rhipicephalus linnaei as Vector
Source: Transbound Emerg Dis. 2025 Jul 3;2025:5542334. doi: 10.1155/tbed/5542334 (PMC12245514; doi:10.1155/tbed/5542334)
Supplement: Supporting Information 4 — Sequences obtained for A. platys 16S gene [file 5542334.f4.docx]

BLASTn comparisons of the isolates from the current study with *Anaplasma platys* sequences from GenBank (June/2024)

| **Isolate** | **Origin** | **Gene** | **Query cover (%)** | **E-value** | **Identity^*^ (%)** | **Closest Match (Acc. Number)** | **Host** | **Location** |
| --- | --- | --- | --- | --- | --- | --- | --- | --- |
| D260 | San Cristóbal | *16S rRNA* | 31 | 5e-26 | 100 | KX082898 | *Canis lupus familiaris* | Angola |
| D424 | San Cristóbal | *16S rRNA* | 100 | 0 | 99.72 | KP903291 | *Canis lupus familiaris* | Cuba |
| D610 | San Cristóbal | *16S rRNA* | 100 | 0 | 99.72 | KP903291 | *Canis lupus familiaris* | Cuba |
| D618 | San Cristóbal | *16S rRNA* | 100 | 0 | 100 | KP903291 | *Canis lupus familiaris* | Cuba |
| ID216 | Isabela | *16S rRNA* | 98 | 0 | 100 | KP903291 | *Canis lupus familiaris* | Cuba |
| SD020 | Santa Cruz | *16S rRNA* | 97 | 0 | 100 | AF478131 | *Rhipicephalus sanguineus* s.l. | Congo |
| D424 | San Cristóbal | *GltA* | 31 | 0 | 99.73 | KT357368 | *Canis lupus familiaris* | Mexico |
| D618 | San Cristóbal | *GltA* | 47 | 0 | 100 | MW450810 | *Canis lupus familiaris* | Grenada |
| SD020 | Santa Cruz | *GltA* | 44 | 0 | 100 | KP903288 | *Canis lupus familiaris* | Cuba |

*the percentage of the nucleotides that are the same between the two sequences
