# Supplementary material for: First Report of Anaplasma phagocytophilum in Galapagos: High Prevalence in Dogs and Circumstantial Evidence for the Role of Rhipicephalus linnaei as Vector
Source: Transbound Emerg Dis. 2025 Jul 3;2025:5542334. doi: 10.1155/tbed/5542334 (PMC12245514; doi:10.1155/tbed/5542334)
Supplement: Supporting Information 5 — BLAST comparisons between the obtained sequences with Rhipicephalus linnaei from GenBank (August, 2024) [file 5542334.f5.docx]

BLASTn comparisons of the isolates from the current study with *Anaplasma phagocytophilum* *GroEL* gene sequences from GenBank (November/ 2024)

| **Isolate** | **Origin** | **Sequence length (bp)** | **Query cover (%)** | **E-value** | **Identity^*^ (%)** | **Closest Match (Acc. Number)** | **Host** | **Location** |
| --- | --- | --- | --- | --- | --- | --- | --- | --- |
| D003 | San Cristóbal | 366 | 99 | 5e-174 | 97.07 | MF372783 | *Erinaceus roumanicus* | Hungary |
| D010 | San Cristóbal | 366 | 99 | 5e-174 | 97.07 | AF478558 | *Capreolus capreolus* | Slovenia |
| D132 | San Cristóbal | 366 | 99 | 5e-174 | 97.07 | MW366836 | *Haemaphysalis concinna* | Hungary |
| D268 | San Cristóbal | 366 | 99 | 5e-174 | 97.07 | MF372783 | *Erinaceus roumanicus* | Hungary |
| D635 | San Cristóbal | 366 | 99 | 5e-174 | 97.07 | HQ629907 | *Ixodes ricinus* | Russia |
| ID004 | Isabela | 366 | 99 | 5e-174 | 97.07 | MN093180 | *Ixodes ricinus* | Netherlands |
| ID029 | Isabela | 366 | 100 | 3e-176 | 97.34 | MF372783 | *Erinaceus roumanicus* | Hungary |
| ID046 | Isabela | 366 | 100 | 2e-174 | 97.07 | MF372783 | *Erinaceus roumanicus* | Hungary |
| ID211 | Isabela | 366 | 100 | 7e-173 | 96.81 | MN093180 | *Ixodes ricinus* | Netherlands |
| FD001 | Floreana | 366 | 100 | 3e-176 | 97.34 | OQ319104 | *Canis lupus familiaris* | Ethiopia |
| SD071 | Santa Cruz | 366 | 100 | 3e-176 | 97.34 | KU712132 | *Vulpes vulpes* | Germany |
| SD092 | Santa Cruz | 366 | 100 | 3e-171 | 96.54 | KU712132 | *Vulpes vulpes* | Germany |
| SD118 | Santa Cruz | 375 | 99 | 5e-174 | 97.07 | ON186490 | *Felis silvestris* | Hungary |
| SD290 | Santa Cruz | 530 | 100 | 7e-168 | 96.01 | ON186492 | *Sciurus vulgaris* | Hungary |

*the percentage of the nucleotides that are the same between the two sequences
